# Acute Effects of Four Major Trace Amines on Zebrafish Behavioral, Neurochemical, and Neuroendocrine Responses

**DOI:** 10.1111/jnc.70116

**Published:** 2025-06-06

**Authors:** Thalia M. Quintanilha, Pietra M. Costa, Ana L. S. Cardoso, Gabrieli S. Battú, Leonardo M. Bastos, Bruno P. dos Santos, Talise E. Müller, Tiago F. de Oliveira, Angelo Piato, Allan V. Kalueff, Murilo S. de Abreu

**Affiliations:** ^1^ Graduate Program in Health Sciences Federal University of Health Sciences of Porto Alegre Porto Alegre Brazil; ^2^ Federal University of Health Sciences of Porto Alegre Porto Alegre Brazil; ^3^ Laboratory of Psychopharmacology and Behavior (LAPCOM), Department of Pharmacology, Institute of Basic Health Sciences Federal University of Rio Grande do Sul Porto Alegre Brazil; ^4^ Institute of Experimental Medicine, Almazov National Medical Research Centre, Ministry of Healthcare of Russian Federation St. Petersburg Russia; ^5^ Institute of Translational Biomedicine, St. Petersburg State University St. Petersburg Russia; ^6^ Suzhou Municipal Key Laboratory of Neurobiology and Cell Signaling, School of Science Xi'an Jiaotong‐Liverpool University Suzhou China; ^7^ Department of Biosciences and Bioinformatics, School of Science Xi'an Jiaotong‐Liverpool University Suzhou China

**Keywords:** acetylcholine, anxiety, behavior, cortisol, trace amines, zebrafish

## Abstract

Trace amines are biologically active compounds endogenously synthesized in the brain in small amounts and structurally resembling biogenic amines. Acting via specific trace amine‐associated receptors (TAARs), they induce robust behavioral and physiological effects in humans and animals. However, although TAAR ligands have recently been suggested as novel putative anxiolytics, their central effects and evolutionary conservation of activity remain poorly understood. Here, we evaluated the acute effects of four major trace amines (beta‐phenylethylamine, tryptamine, tyramine, and octopamine) on zebrafish anxiety‐like and social (shoaling) behavior, as well as neurochemical and neuroendocrine (cortisol) responses. Beta‐phenylethylamine, at a low concentration (12 μg/L), caused overt anxiolytic‐like effects and reduced brain acetylcholine levels; at a high concentration (1000 μg/L) increased zebrafish anxiety‐like behavior and whole‐body cortisol levels. Acute tryptamine exposure (7 mg/L) evoked an anxiogenic‐like effect, reduced shoaling and social interaction, and elevated brain acetylcholine and whole‐body cortisol. Acute exposure to tyramine (15 μg/L) and octopamine (125, 500, and 1500 μg/L) induced similar anxiogenic‐like effects, accompanied by increased whole‐body cortisol without altering brain acetylcholine levels. Collectively, these findings not only emphasize the important role of trace amines in brain and behavior but support the growing complexity of their CNS effects in vivo across taxa and highlight the relevance of zebrafish models for drug screening based on targeting brain TAARs.

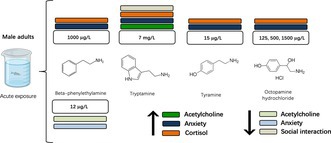

## Graphical abstract

A trace amine beta‐phenylethylamine is anxiolytic and reduces brain acetylcholine at 12 μg/L, but elevates anxiety and cortisol levels at 1000 μg/L. Acute tryptamine at 7 mg/L is anxiogenic, reduces shoaling and social interaction, but elevates brain acetylcholine and cortisol levels. Acute tyramine at 15 μg/L, and octopamine at 125, 500, and 1500 μg/L, induce anxiogenic‐like effects and increase cortisol levels without affecting brain acetylcholine.

**Abbreviations**
AMPA: α‐amino‐3‐hydroxyl‐5‐methyl‐4‐isoxazole‐propionate
CNS: central nervous system
DMSO: dimethyl sulfoxide
ESI: electrospray ionization
KW: Kruskal‐Wallis (test)
LC–MS/MS: liquid chromatography‐mass spectrometry/mass spectrometry
MAO: monoamine oxidase
MRM: multiple reaction monitoring
PBS: phosphate buffered saline
RRID: Research Resource Identifier
TAARs: trace amine‐associated receptors
TrkB: tropomyosin receptor kinase B

## 1 Introduction

Trace amines are biologically active compounds that are endogenously synthesized in small (< 10 ng/g) amounts in the brain and structurally resemble biogenic amines, such as serotonin and dopamine (Fehler et al. 2010; Lindemann and Hoener 2005; Gainetdinov et al. 2018). P‐tyramine, β‐phenylethylamine, tryptamine, and octopamine represent common trace amines (Gainetdinov et al. 2018). The trace amines act via their specific trace amine‐associated receptors (TAARs) (Gainetdinov et al. 2018; Borowsky et al. 2001; Bunzow et al. 2001), which vary widely, from 6 in humans (TAAR1, TAAR2, TAAR5, TAAR6, TAAR8, and TAAR9) to 15 in mice and 112 in zebrafish ( *Danio rerio* ) (Li et al. 2015; Galstyan, Krotova, et al. 2025).

Beta‐phenylethylamine acts via TAAR1 (Borowsky et al. 2001) and is widespread throughout the central nervous system (CNS) in humans (Philips et al. 1978) and mammals (Durden et al. 1973). In rodents, it is synthesized within nigrostriatal and mesolimbic dopamine regions (Paterson et al. 1990) and evokes multiple behavioral effects, including anxiety‐like behavior, decreased social interaction (Lapin 1990) and stereotyped behavior (Borison et al. 1977; Ortmann et al. 1984; Barroso and Rodriguez 1996) (also see similar primate findings; Tinklenberg et al. 1978). Tryptamine is synthesized in the brain by decarboxylation of L‐tryptophan and acts via TAAR2 (Gainetdinov et al. 2018; Borowsky et al. 2001; Bunzow et al. 2001) and serotonergic (e.g., 5HT1A and 5HT2A) receptors (Fantegrossi et al. 2008; Woolley and Shaw 1962; Kargbo 2023), evoking various CNS effects. For example, it increases rat locomotor and vertical activity following bilateral intra‐accumbal injections (Marien et al. 1987) and reverses reserpine‐induced depression‐like states in rabbits (Tedeschi et al. 1959). Tryptamine‐based drug molecules (e.g., psilocybin) induce anxiolytic‐ and antidepressant‐like effects in rodents (Sekssaoui et al. 2024) and have recently been approved for treatment‐resistant depression and major depression clinically (Kargbo 2023; Raison et al. 2023; Goodwin et al. 2022).

Tyramine, another common trace amine, is produced via the decarboxylation of tyrosine and degraded by several enzymes, including monoamine oxidase (MAO) (Gillman 2018; Andersen 1977). Tyramine has high affinity for binding TAAR1 and TAAR2 (Gainetdinov et al. 2018) and exhibits multiple neuromodulatory and other physiological effects in humans and animals (Andersen 1977; Nagaya et al. 2002). For example, its intake is associated with migraine onset (Costa and Glória 2003) and with mood and cognitive deficits and switch from hypomania to depression, suggesting that affective behaviors may be particularly susceptible to this trace amine (Pickar et al. 1979). In turn, octopamine is synthesized in the brain from tyramine and also plays an important CNS role (Berry 2004; Burchett and Hicks 2006; Axelrod and Saavedra 1977). For example, octopamine intraventricular infusion evokes rat depression‐like (Chance et al. 1985) and hypolocomotor phenotypes (Delacour and Guenaire 1983; David et al. 1982). Aberrant tyramine and octopamine neurotransmission has also been associated with a wide range of clinical CNS disorders (Zucchi et al. 2006), as they are both reduced in depression (Sandler et al. 1979), whereas tyramine levels are elevated in Parkinson's patients (D'Andrea et al. 2019), and octopamine levels are lower in patients with encephalopathy (Cuilleret et al. 1980).

Mounting clinical and preclinical studies suggest trace amines as promising modulators of stress‐ and anxiety‐related behaviors (Wolinsky et al. 2007; Rutigliano et al. 2019; Liu et al. 2024) which can be corrected by TAARs ligands, a recently proposed new putative class of anxiolytic drugs (Alnefeesi et al. 2024). In addition to monoaminergic and trace‐aminergic signaling, the central cholinergic system potently modulates various brain functions, including anxiety‐ and depression‐like behavior (Mineur et al. 2018, 2013, 2022). For example, depressed patients have higher brain levels of acetylcholine than healthy controls (Hannestad et al. 2013; Esterlis et al. 2013), whereas stressed rats display elevated hippocampal acetylcholine (Mark et al. 1996; Mineur et al. 2022). Elevating acetylcholine levels by inhibiting hippocampal acetylcholinesterase in mice induces anxiety‐like and depression‐like behaviors (Mineur et al. 2013), whereas oxotremorine (a muscarinic acetylcholine receptor agonist) attenuates anxiety induced by chronic stress (Di Liberto et al. 2017).

Complementing rodent models, the zebrafish ( *Danio rerio* ) is rapidly becoming a powerful model species in neuroscience and neurochemistry (Costa et al. 2023), including trace amine‐ (Galstyan, Krotova, et al. 2025) and cholinergic neurotransmission research (Mueller et al. 2004; Clemente et al. 2004). For instance, zebrafish have 20%–48% orthologous genes, similar to those of mammalian TAAR1 receptors (Grandy 2007). Recent studies have demonstrated CNS effects of novel trace amine‐like molecules in zebrafish models, as acute 24H‐NBOMe(F) and 34H‐NBOMe(F) treatment reduce zebrafish despair‐like behavior (Demin et al. 2022), while chronic exposure to 24H‐NBOMe(F) and 34H‐NBOMe(F) evokes anxiolytic‐like effects in the novel tank test with lowered brain norepinephrine levels, further supporting the potential of zebrafish for preclinical screening of small psychoactive trace amine‐related molecules (Ilyin et al. 2024). Likewise, although acute exposure to cholinergic drugs nicotine, varenicline, and arecoline produces anxiolytic‐like effects in zebrafish (Serikuly et al. 2020; Levin et al. 2007), donepezil (an acetylcholinesterase inhibitor facilitating acetylcholine action) increases anxiety‐like behavior and whole‐body cortisol levels (Giacomini, Bueno, et al. 2020).

In addition to high genetic and physiological homology to humans in general (Howe et al. 2013; Briggs 2002), zebrafish possess evolutionarily conserved, shared neuroanatomy and neurochemistry of both the trace amine‐ (Galstyan, Krotova, et al. 2025) and cholinergic systems (Mueller et al. 2004), and may therefore be used to assess the role of both systems and their interplay in stress and stress‐related behavioral deficits. Recognizing the growing value of zebrafish models for studying the trace‐aminergic and cholinergic modulation of CNS functions and complex behaviors, here we examined the effects of four common trace amines (β‐phenylethylamine, tryptamine, tyramine, and octopamine) on zebrafish anxiety‐like and social behaviors, central acetylcholine levels, and whole‐body cortisol.

## 2 Methods

### 2.1 Animals

A total of 284 male mature adults (4–6 months old) zebrafish of the wild‐type short‐fin outbred strain were obtained from a commercial supplier (Delphis, Porto Alegre, Brazil). Fish were acclimated to the laboratory environment for 1 month and housed (3 fish/L) in 13‐L tanks (15 width × 35 depth × 25 height, cm) equipped with external biological filters (Ocean Tech HF‐100, Brazil) under constant aeration and a light/dark (14 h light:10 h dark) cycle, with lights on at 7:00 am. Water temperature was maintained at 27.2°C ± 0.5°C; pH 6.8 ± 0.15, with dissolved oxygen kept at 6 ± 0.15 mg/L, total ammonia at < 0.01 mg/L, according to established standards of zebrafish husbandry (Avdesh et al. 2012). All fish were fed twice a day with commercial flake food (Alcon Basic, MEP 200 Complex, Brazil), following accepted standards of zebrafish husbandry. The selection of outbred fish for this study was based on the relevance of translational research to better mimic the genetic heterogeneity of clinical populations (de Abreu et al. 2022). Animal experimentation reported here was approved by the Institutional Animal Care and Use Committee of the Federal University of Health Sciences of Porto Alegre (Ref. 816/2024) and fully adhered to national and international guidelines on animal experimentation and care and principles of ethical experimentation (de Abreu et al. 2019). The experimental design, data analysis, and presentation fully adhered to the ARRIVE 2.0 (Animal Research: Reporting of In Vivo Experiments) guidelines (Percie du Sert et al. 2020) for reporting animal research and the PREPARE (Planning Research and Experimental Procedures on Animals: Recommendations for Excellence) guidelines (Smith et al. 2018) for planning animal research and testing. The methods were presented following the Research Resource Identifiers (RRIDs).

### 2.2 Experimental Procedures

#### 2.2.1 Acute Effects of β‐Phenylethylamine and Tryptamine

Experiment 1 examined the acute effects of β‐phenylethylamine (CAS: 64‐04‐0, Sigma‐Aldrich, St. Louis, USA) on zebrafish anxiety‐like behavior. Using an online randomization tool (www.randomizer.org), zebrafish (*n* = 13 per group) were randomly allocated into 5 experimental groups: control (intact water, unexposed) and β‐phenylethylamine‐treated (at 12, 120, 300, and 1000 μg/L) via water immersion for 1 h. The 1 h treatment time and immersion administration were selected based on our previous studies on anxiolytic drugs in adult zebrafish (Giacomini et al. 2021; Giacomini, Piassetta, et al. 2020). The concentrations of β‐phenylethylamine used here were selected based on half maximal effective concentration (EC_50_ = ~0.3 μM, ~36 μg/L) for activation of the TAAR12 receptor in zebrafish (Li et al. 2015) and TAAR1 in humans (Lindemann et al. 2005), as well as on our pilot study with this drug. The sample size (*n* = 13) in this study, necessary to obtain reliable data, was estimated based on our previous experience screening drugs in adult zebrafish (Giacomini et al. 2021), as well as prior calculations using the G*Power 3.1 program (Heinrich Heine University Düsseldorf, Germany) for one‐way analysis of variance (ANOVA), effect size of 0.5, alpha of 0.05, power of 0.9, 5 groups, and a total *n* = 70.

Since the novel tank test identified the 12 μg/L concentration of the drug as inducing anxiolytic‐like behavior, we assessed brain levels of acetylcholine, as well as whole‐body cortisol levels. In addition, as the 1000 μg/L concentration of β‐phenylethylamine demonstrated an anxiogenic‐like (e.g., increasing freezing duration), an additional study was performed to examine this aspect in detail, assessing zebrafish behavior in an anxiety‐sensitive shoaling test and social interaction assay. For this, a separate cohort of zebrafish from the same batch was randomly allocated into control and β‐phenylethylamine‐treated (at 1000 μg/L) groups (*n* = 4). The fish were initially treated in the group (4 animals each) for 1 h and then tested in the shoaling test for 5 min, and subsequently individually recorded for 10 s in the social interaction assay (see further). The group size (4 fish shoal) in this study, necessary to obtain reliable data, was estimated based on our previous experience screening drugs in adult zebrafish (Giacomini, Bueno, et al. 2020). At the end of behavioral analyses, zebrafish were euthanized in ice‐cold water (with decapitation after the cessation of opercular movements for > 30 s), and their brains were briefly extracted on ice to assess acetylcholine brain levels. The headless bodies were used to reconfirm sexing by gonadal dissection under a PZ0‐Labimex stereomicroscope (Metrimpex, Budapest, Hungary) and later stored at −80°C in an ultra‐freezer for whole‐body cortisol analyses.

Experiment 2 examined the acute effects of tryptamine (CAS: 61‐54‐1, Sigma‐Aldrich) on zebrafish anxiety‐like behavior and physiology. Zebrafish (*n* = 12 per group) were randomly allocated into 4 experimental groups: control (dimethyl sulfoxide/DMSO 0.011% vol/vol) and tryptamine‐treated (at 1.5, 7, and 15 mg/L) via water immersion for 1 h. The concentrations of tryptamine used here were selected based on half maximal effective concentration (EC_50_ = ~70 μM, ~11.21 mg/L) for activation of TAAR10a in zebrafish (Li et al. 2015). We also performed a pilot study to verify whether the concentration of DMSO 0.011% used in the control group would influence the zebrafish anxiety‐like behavior. As no difference was found between control (DMSO 0.011%) and intact control groups, and compliant with the reduction of animal usage as per 3Rs principles of human bioexperimentation, we used the DMSO 0.011% group as control for all experiments. Similarly, to Experiment 1, zebrafish were individually exposed to 800 mL tryptamine or vehicle for 1 h in the treatment tanks, followed by assessing their behavior in the novel tank test for 6 min.

Since this assay identified the 7 mg/L concentration of the drug as behaviorally active, producing an anxiogenic‐like effect, we conducted an additional study to explore this further by assessing zebrafish behavior in the shoaling and social interaction tests. For this, a separate cohort of zebrafish from the same batch was randomly allocated into control (DMSO 0.011%) and tryptamine‐treated (7 mg/L) groups (*n* = 6). The fish were initially treated in the 4‐fish group for 1 h, tested in the shoaling test for 5 min, and then subsequently individually recorded for 10 s in the social interaction assay (see further). The group size here was estimated based on our previous experience screening drugs in adult zebrafish (Giacomini, Bueno, et al. 2020). As in Experiment 1, at the end of behavioral analyses, zebrafish were euthanized in ice‐cold water and their brains were briefly extracted on ice to assess brain acetylcholine levels and whole‐body cortisol, as well as to reconfirm sexing by gonadal dissection.

#### 2.2.2 Acute Effects of Octopamine and Tyramine

Experiment 3 examined the acute effects of tyramine (CAS: 51‐67‐2) and octopamine hydrochloride (CAS: 770‐05‐8, Sigma‐Aldrich) on zebrafish anxiety‐like behavior and physiology. Zebrafish (*n* = 13 per group) were randomly allocated into 7 experimental groups: control (intact water, unexposed), treated with tyramine (at 5, 15, and 135 μg/L) and with octopamine (at 125, 500, and 1500 μg/L) via water immersion for 1 h. The concentrations of tyramine used here were selected based on half maximal effective concentration (EC_50_ = ~1 μM, ~137.18 μg/L) for the activation of human and mouse TAAR1 (Lindemann et al. 2005), and the concentrations of octopamine were based on EC_50_ of ~10 μM (~1.89 mg/L) for human TAAR1 (Lindemann et al. 2005). As in Experiment 1 and 2, at the end of behavioral analyses, zebrafish were euthanized in ice‐cold water to assess whole‐body cortisol and to reconfirm sexing by gonadal dissection.

The respective trace amines levels in exposure solution samples were reconfirmed by liquid chromatographic system coupled to a triple quadrupole mass spectrometer (LC–MS/MS), model LCMS‐8045 (Shimadzu, Kyoto, Japan), showing the actual concentrations of β‐phenylethylamine 12 μg/L (12.3 ± 3.22 μg/L), 120 μg/L (126 ± 11 μg/L), 300 μg/L (280 ± 44 μg/L), and 1000 μg/L (1004 ± 114 μg/L), tryptamine 1.5 mg/L (1.53 ± 0.11 μg/L), 7 mg/L (7 ± 0.22 mg/L), 15 mg/L (15.57 ± 0.43 mg/L), tyramine 5 μg/L (5.1 ± 0.6 μg/L), 15 μg/L (14.4 ± 1 μg/L), 135 μg/L (135.8 ± 0.8 μg/L) and octopamine 125 μg/L (123.1 ± 6.66 μg/L), 500 μg/L (495 ± 54.8 μg/L), 1500 μg/L (1512 ± 134.5 μg/L).

### 2.3 Behavioral Testing

Behavioral testing was performed in the animal holding room (to avoid transfer and manipulation stress) between 8.00 am and 11.00 am, recording fish behaviors by C920 cameras (Logitech Inc., Romanel‐sur‐Morges, Switzerland). On the day of the experiment, the fish remained unfed to avoid a concomitant effect of feeding on behavioral endpoints and were randomly allocated to behavioral testing tanks to avoid experimental bias. Behavioral, neurochemical, and endocrine assays, as well as subsequent offline data analyses, were performed by investigators blinded to the experimental groups (each tank was coded by a researcher who did not participate in the experiments).

Zebrafish were individually exposed for 1 h to treatment in the glass tank (10 width × 10 depth × 15 height, cm) with 800 mL of drug or vehicle solution prior to assessing their behavioral response in the novel tank test for 6 min. Its apparatus was a glass tank (24 width × 8 depth × 20 height, cm) filled with facility water to a height of 18 cm. During the test, we recorded zebrafish individually with a C920 camera and subsequently analyzed data offline using ANY‐maze software (Stoelting Co, Wood Dale, USA), assessing the total distance traveled, freezing frequency (immobility > 2 s) and duration, as well as the time spent at the top of the tank, based on the body center position, as in (Kysil et al. 2017).

Fish were also treated with drugs or vehicle in 4 fish groups for 1 h, tested in the shoaling test for 5 min, after which they were individually assessed in the social interaction test. For the latter test, fish were transferred individually to the glass testing tank (30 width × 10 depth × 15 height, cm), positioned between two other tanks, one empty and the other containing a group of 15 conspecifics (Giacomini et al. 2016). In this test, fish acclimated for 30 s to the tank, and their behavior was then recorded for 10 s, with a subsequent off‐line analysis using the ANY‐maze software to assess the time spent near conspecifics. Shoaling test videos were further analyzed using the Image‐J‐1.49 software (National Institutes of Health, NIH, Bethesda, USA), and the shoaling phenotypes were assessed using screenshots made every 20 s over the test period (with a total of 15 screens shots per group), to calculate the dispersion of the group as the shoal size (Canzian et al. 2017). At the end of behavioral analyses, zebrafish were euthanized in ice‐cold water (followed by decapitation after the cessation of breathing movements for > 30 s) and their brains quickly extracted on ice for neurochemical analyses, and headless bodies—for whole‐body cortisol analyses and to reconfirm sexing by gonadal dissection under the microscope. The whole‐brain and whole‐body samples were stored in the ultra‐freezer at −80°C for further analyses.

### 2.4 Brain Acetylcholine Analyses

Brain samples were thawed, weighed, placed in test tubes with 10 μL of 0.1% formic acid solution per 1 mg/brain weight, and homogenized in the microtube with a plastic pestle for 1 min. In 50 μL of homogenized sample, we added 930 μL of pure acetone and 20 μL of the *d*‐dopamine standard (250 ng/mL, Sigma‐Aldrich) and centrifuged for 6 min (at 9000 rpm, 5610 rcf), after which the supernatant was placed to evaporate in the laminar flow hood until full evaporation and stored at −80°C in the ultra‐freezer. Neurochemical analyses were performed using the liquid chromatographic system coupled to an 8045‐Shimadzu triple quadrupole mass spectrometer (LC–MS/MS, Shimadzu Inc., Kyoto, Japan). On the day of analyses, brain samples were resuspended in ultrapure H_2_O (50 μL), and the mass spectrometer was operated using electrospray ionization (ESI), with the acquisition of all analytes made in the positive mode and settings used for analyses as heat block temperature 400°C, capillary voltage 4.5 kV, nebulizer gas (N_2_) flow 1.7 L/min, drying gas (N_2_) flow 10 L/min, desolvation line temperature 250°C, and collision‐induced dissociation gas pressure 230 kPa. Multiple reaction monitoring (MRM) mode was used for data acquisition, with two MRM transitions for each analyte—one for quantification and the other for qualification. The MRM transitions (m/z) and collision energy (V) were: acetylcholine (147.1–84.0/17 and 147.1–130.1/20).

For neurochemical analyses, the chromatographic separation was performed with a Shimpack GISS C18 column (100 × 2.1 mm, 1.9‐μm particle size) from Shimadzu Inc. (Japan). The mobile phases were composed of (A) ultra‐pure water with formic acid (0.1%, *v/v*) and (B) acetonitrile with formic acid (0.1%, *v/v*). The column was heated at 30°C, with a mobile phase flow of 0.3 mL/min. The chromatographic run was performed using a gradient as follows: 0–1 min, 0% of B; 1–6 min, 0%–100% of B; 6–7 min, 100% of B; 7–7.1 min, 100%–0% of B; 7.1–15 min, 5% of B, totaling 15 min. All data were collected and analyzed using the LabSolutions software (Shimadzu Inc.).

### 2.5 Whole Body‐Cortisol Analysis

Fish were homogenized by a Dremel‐300 tissue homogenizer (Dremel Inc., Rancine, USA) in phosphate‐buffered saline (0.5 g weight in 2 mL PBS g) for 1 min for their whole‐body cortisol extraction with diethyl ether, as described previously (de Abreu et al. 2014). Cortisol levels were analyzed using a commercially available cortisol enzyme‐linked immunosorbent assay kit (ELISA, Monobind Inc., Lake Forest, USA) for human serum or plasma (DCO: 1353, product code: 3625‐300). The detection accuracy was tested by calculating the recoveries from standard samples containing 1, 4, 10, 20, and 50 ng/mL cortisol, with a mean detection of 95%. All cortisol values were adjusted for recovery (cortisol value = measured value × 1.05) and normalized based on the weight of the respective whole‐body samples, expressed as absolute cortisol concentrations (ng/g body weight).

### 2.6 Data Analysis

Data normality was assessed by the Kolmogorov‐Smirnov test. In all experiments, the novel tank data were analyzed by the Kruskal‐Wallis (KW) test, followed by Dunn's post hoc test for significant KW data. Shoal area and acetylcholine data were analyzed using an unpaired t‐test, while social interaction was assessed using the Wilcoxon‐Mann–Whitney U‐test. The whole‐body cortisol data were analyzed by one‐way ANOVA (factor: drug), followed by Dunnett's post hoc test (Experiment 1), U‐test (Experiment 2) and KW test, followed by Dunn's post hoc test (Experiment 3). In all analyses, groups were compared with the control group, and *p‐values* were set at < 0.05. Behavioral tests were performed by highly trained experimenters in zebrafish assays, with intra/inter‐rater reliability > 0.9, as assessed by Spearman correlation. All data were included in analyses, without removing outliers. Statistical analyses were performed using GraphPad Prism 9.5 statistical software (GraphPad Software, San Diego, USA).

## 3 Results

**FIGURE 1:**
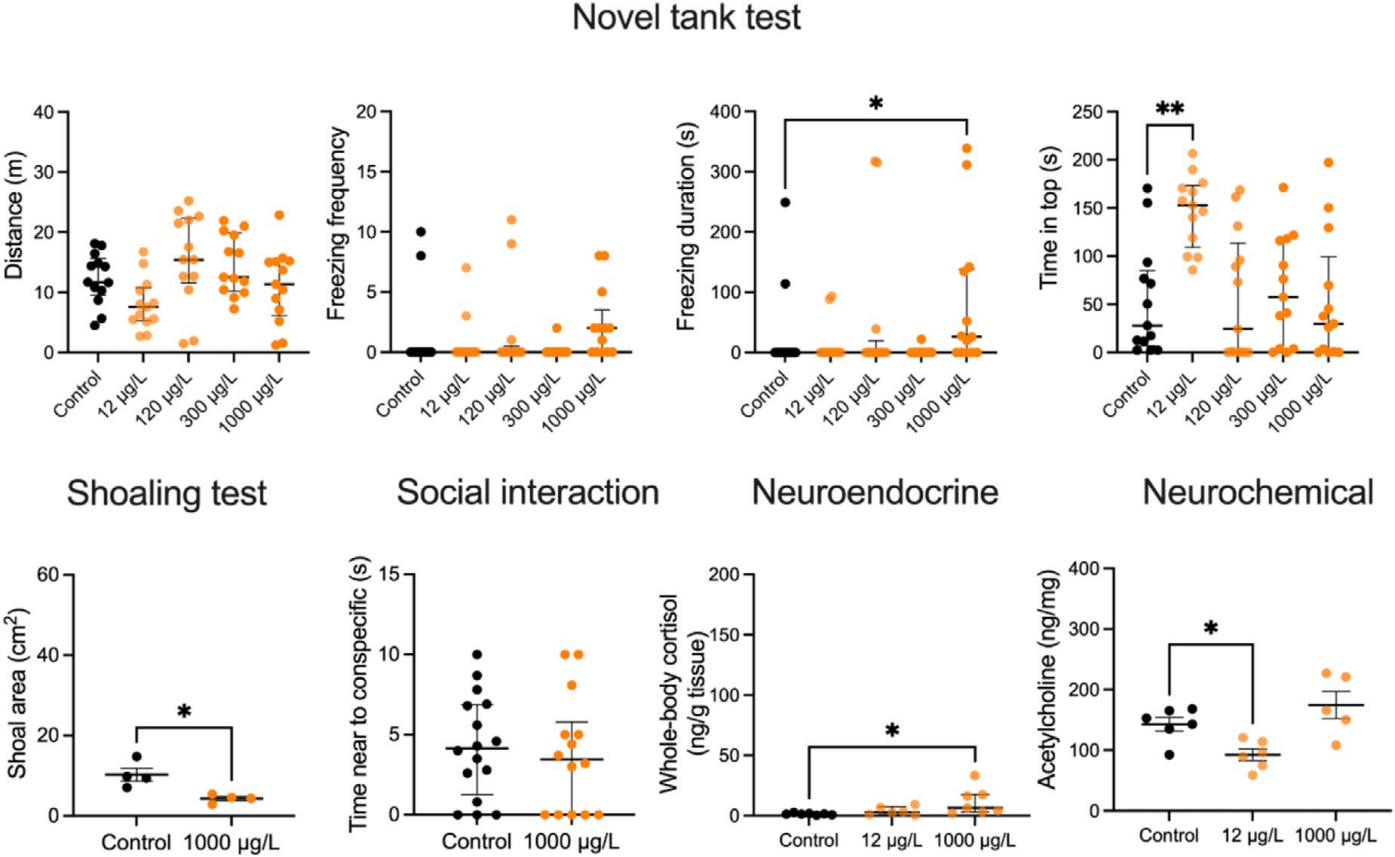
Effects of acute 1‐h β‐phenylethylamine exposure on zebrafish behavior in the 6‐min novel tank test (top panel, *n* = 13 per group), shoaling (*n* = 4 per cohort) and social interaction tests (control *n* = 16 and β‐phenylethylamine *n* = 14), as well as whole‐body cortisol levels (control and β‐phenylethylamine 1000 μg/L *n* = 7, and 12 μg/L *n* = 6), and brain acetylcholine (control and β‐phenylethylamine 12 μg/L *n* = 6, and 1000 μg/L *n* = 5, bottom panel). The individual behavioral data are presented as median ± interquartile range, and shoaling test, acetylcholine, and whole‐body cortisol data as mean ± SEM. *p < 0.05, **p < 0.01, Dunn's post hoc test for significant Kruskal‐Wallis data or Dunnett test for significant one‐way ANOVA data.

Experiment 1 showed significant group/treatment effects for distance traveled (*H*_5,65_ = 13.05, *p* = 0.0011), freezing frequency (*H*_5,65_ = 10.63, *p* = 0.031) and freezing duration (*H*_5,65_ = 12.08, *p* = 0.0168), as zebrafish treated with 1000 μg/L β‐phenylethylamine demonstrated longer freezing compared to the control group (*p* = 0.0412), reflecting anxiety‐like behavior (Figure 1). There were also significant treatment effects for time in top (*H*_5,65_ = 17.99, *p* = 0.0012), as zebrafish treated with 12 μg/L of this drug demonstrated longer time in top than the control (*p* = 0.0064), indicative of an anxiolytic‐like effect (Figure 1). Zebrafish exposed to β‐phenylethylamine at 1000 μg/L also showed reduced shoal area (*p* = 0.0121), with no significant effect of this drug on social interaction (*p* = 0.64, NS). There were also significant treatment effects for whole‐body cortisol levels (*F*_2,17_ = 4.19, *p* = 0.0332) and brain acetylcholine levels (*F*_2,14_ = 8.03, *p* = 0.0048), as zebrafish treated with 1000 μg/L increased the whole‐body cortisol versus the control group (*p* = 0.0235), whereas 12 μg/L also decreased brain acetylcholine levels (*p* = 0.0435 vs. control, Figure 1).

**FIGURE 2:**
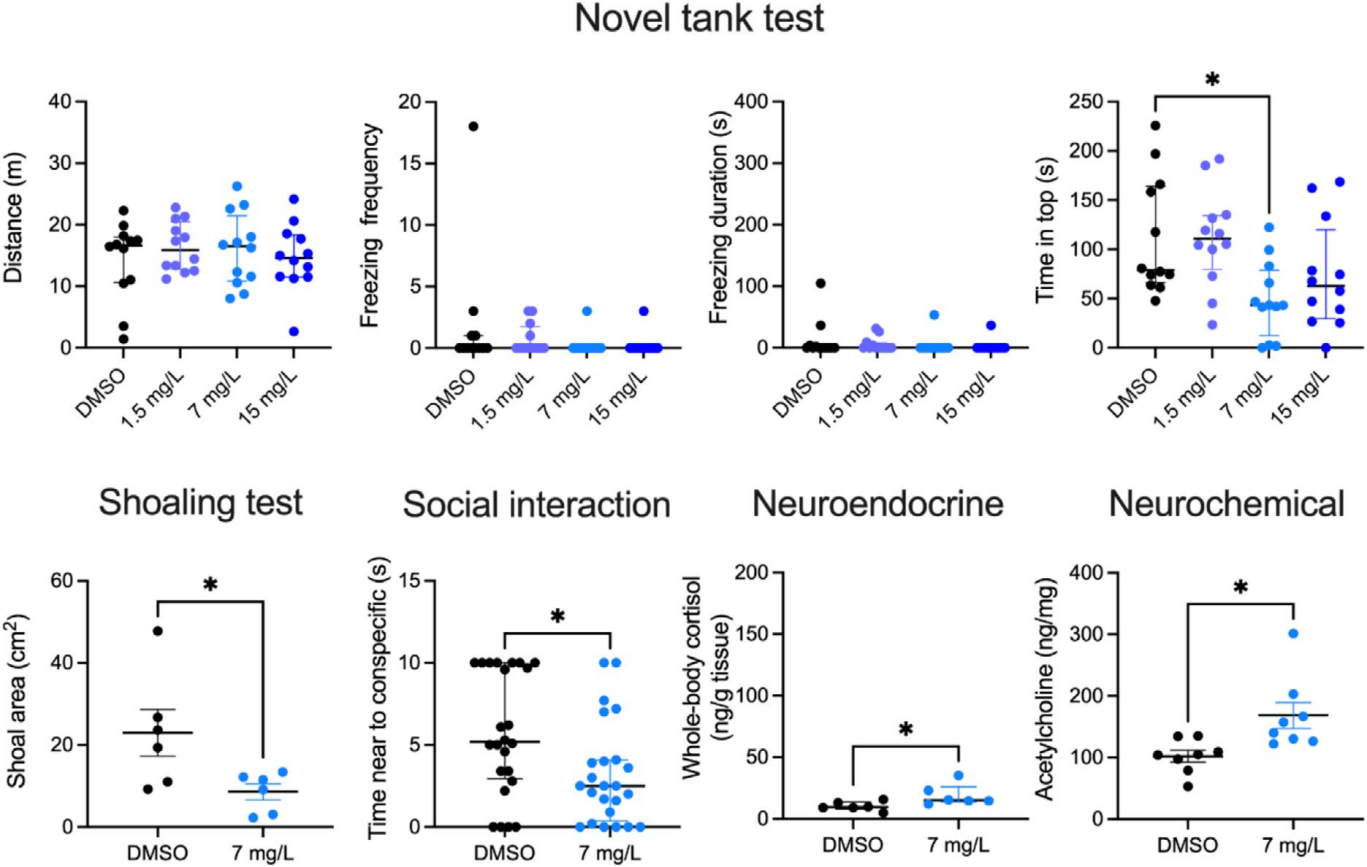
Effects of acute 1‐h tryptamine exposure on zebrafish behavioral responses in the novel tank (*n* = 12 per group), shoaling (*n* = 6 per cohort) and social interaction tests (*n* = 24). Individual behavioral and whole‐body cortisol data (*n* = 6) are presented as median ± interquartile range, and shoaling test‐ and brain acetylcholine data (*n* = 8) as mean ± SEM. *p < 0.05, Dunn's post hoc test for significant Kruskal‐Wallis data or *U*‐test, where applicable.

Experiment 2 found a significant treatment effect for time in top (*H*_4,48_ = 11.38, *p* = 0.0098), as zebrafish treated with 7 mg/L tryptamine spent more time there versus control (*p* = 0.0184, Figure 2). In contrast, there were no significant treatment effects for distance traveled (*H*_4,48_ = 0.74, *p* = 0.86, NS), freezing episodes (*H*_4,48_ = 4.02, *p* = 0.25, NS) and freezing duration (*H*_4,48_ = 3.73, *p* = 0.29, NS) in all groups tested. However, zebrafish treated with 7 mg/L tryptamine showed reduced shoal area (*p* = 0.0385) and time near the conspecifics (*p* = 0.0163), but elevated brain acetylcholine (*p* = 0.013) and whole‐body cortisol levels (*p* = 0.041) compared to the control group (Figure 2).

**FIGURE 3:**
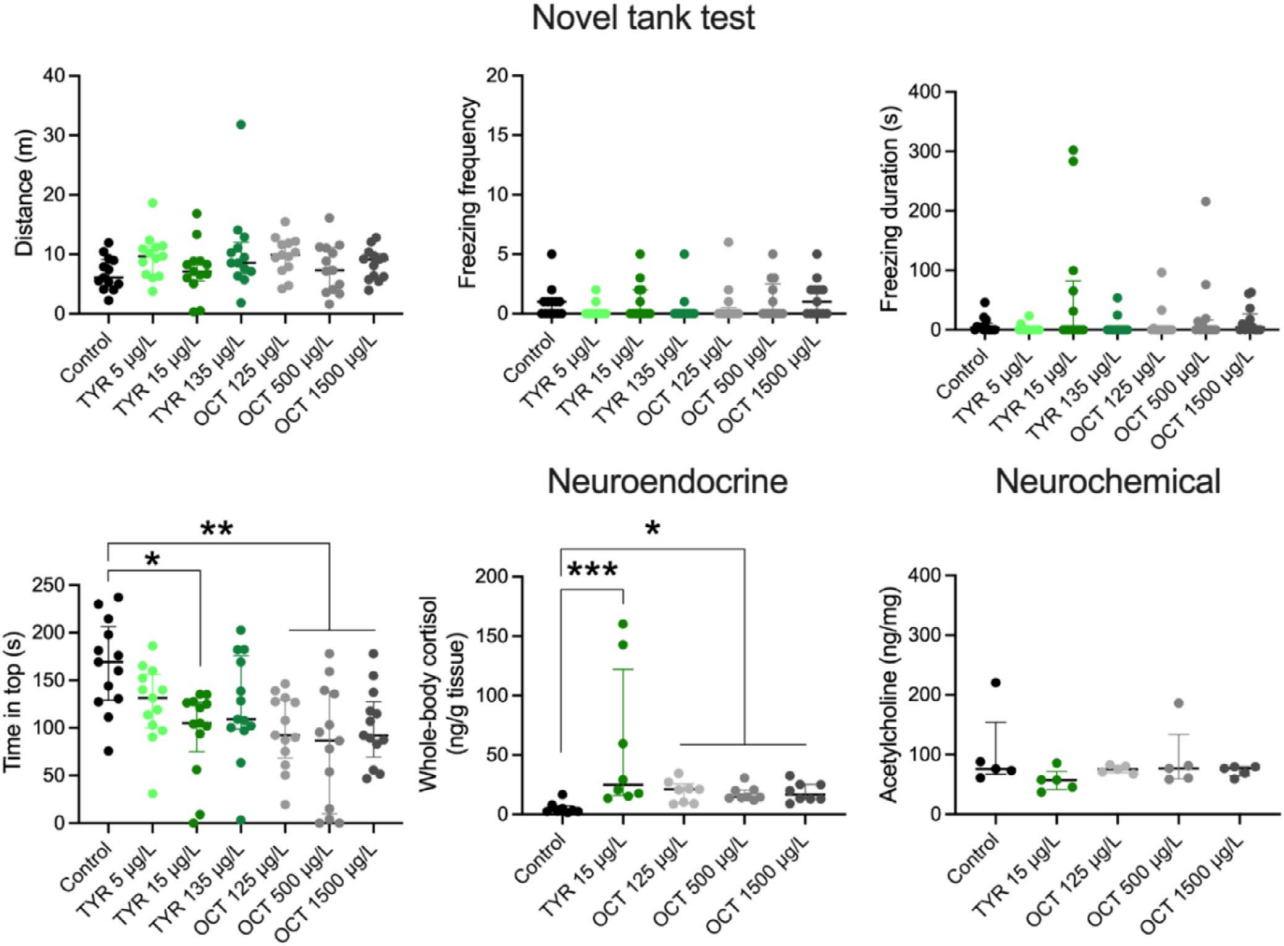
Effects of acute 1‐h tyramine and octopamine exposure on zebrafish behavioral responses in the novel tank test (*n* = 13 per group), whole‐body cortisol (*n* = 8), and brain acetylcholine levels (*n* = 5). Data are presented as median ± interquartile range. *p < 0.05, **p < 0.01, and ***p < 0.001 Dunn's post hoc test for significant Kruskal‐Wallis data.

Experiment 3 revealed a significant treatment effect for the time in top (*H*_7,91_ = 20.53, *p* = 0.0022), as zebrafish treated with 15 μg/L of tyramine (*p* = 0.0104) and octopamine at 125 (*p* = 0.0066), 500 (*p* = 0.0014) and 1500 μg/L (*p* = 0.0079) demonstrated lower time at top versus control group, representing an anxiety‐like behavior (Figure 3). There were no significant effects of these drugs for distance traveled (*H*_7,91_ = 8.79, *p* = 0.18, NS), freezing episodes (*H*_7,91_ = 8.66, *p* = 0.19, NS), and freezing duration (*H*_7,91_ = 8.03, *p* = 0.24, NS). We also found a significant treatment effect for whole‐body cortisol levels (*H*_5,40_ = 17.44, *p* = 0.0016), as zebrafish treated with 15 μg/L of tyramine (*p* = 0.0002) and octopamine at 125 (*p* = 0.0145), 500 μ (*p* = 0.0363) and 1500 μg/L (*p* = 0.0310) increased the whole‐body cortisol levels compared to control group (Figure 3). In contrast, there were no significant effects of these drugs on brain acetylcholine levels (*H*_5,25_ = 5.51, *p* = 0.24, NS).

## 4 Discussion

The present study is the first report of overt behavioral, neuroendocrine, and neurochemical (cholinergic) effects in zebrafish following acute exposure to four major trace amines. We found dose‐dependent CNS effects of acute β‐phenylethylamine, at low concentrations causing anxiolytic‐like effects in adult male zebrafish. Consistent with this, β‐phenylethylamine in rodents ameliorates depression‐like and other CNS deficits (e.g., decreased hippocampal brain‐derived neurotrophic factor and TrkB expression) evoked by corticosterone injections (Lee et al. 2020). Treating with β‐phenylethylamine or its precursor L‐phenylalanine also improves mood in depressed patients (Sabelli et al. 1989; Sabelli and Javaid 1995). In contrast, a high concentration of β‐phenylethylamine (1000 μg/L) increased anxiety‐like behavior in zebrafish, prolonging freezing duration in the novel tank test and decreasing the shoal area in the shoaling test—both phenotypes associated with anxiety‐like states (Egan et al. 2009; Kalueff et al. 2013). Interestingly, the inactivation of the MAO B gene in mice increases brain levels of β‐phenylethylamine, and *MAOB*‐deficient mice display increased stress reactivity (Grimsby et al. 1997), further supporting the role of β‐phenylethylamine in stress response. Acute treatment with 10 mg/kg β‐phenylethylamine is similarly anxiogenic in the mouse elevated plus‐maze (Lapin 1993) and light/dark box tests, also reducing social interaction (Lapin 1990) and promoting stereotypies (Ryu et al. 2021).

Moreover, similar biphasic effects have been demonstrated in zebrafish by various other CNS drugs, as the exposure to intermediate concentrations of alcohol (0.5%) exerts a stimulant effect manifesting as a slight elevation of swim speed, a robust increase in turning, temporal variability in swim speed and turning, and diminished frequency of staying immobile, while high concentrations (1%) elicit an opposite, sedative effect in zebrafish larvae (Tsang et al. 2019). Butaclamol (a dopaminergic antagonist) increases larval locomotion at low‐to‐intermediate concentrations and reduces it at high concentrations (Irons et al. 2013). Similarly, adult fish display a U‐shaped dose–response pattern in locomotor activity when exposed to ethanol (Gerlai et al. 2000), and in whole‐body cortisol levels when exposed to diazepam (de Abreu et al. 2014).

Furthermore, we also found anxiogenic‐like and anti‐social effects for acute tryptamine (7 mg/L) in zebrafish tested here. While intravenously administered tryptamine induces serotonin toxicity syndrome‐like head‐weaving and hindlimb abduction in mice (Yamada et al. 1987), rats treated with *N*,*N*‐dimethyltryptamine, a hallucinogenic tryptamine derivative, display similar anxiogenic‐like responses in the open field test, but not in the social interaction test (Cameron et al. 2018). Acute tyramine (15 μg/L) and octopamine (125, 500, and 1500 μg/L) also evoked overt anxiogenic‐like effects in male zebrafish here. Tyramine is a vasoactive amine that can trigger a migraine attack in susceptible individuals (Costa and Glória 2003), elevate blood pressure nociception, nausea, visual deficits (Astudillo et al. 2024), sympathetic activation, and, occasionally, anxiety symptoms clinically (Ogedegbe et al. 2008; Lim et al. 2021; McCabe 1986). Octopamine has also been shown to modulate CNS and behavior, and its intraventricular infusions cause various neurological deficits in rodents, also decreasing brain norepinephrine, dopamine, and serotonin levels (Chance et al. 1985).

Analyzing fish neurochemical profiles, we found that a low 12 μg/L concentration of β‐phenylethylamine reduces brain acetylcholine levels (Figure 1). Interestingly, in rats this drug stimulates striatal acetylcholine release through the activation of the α‐amino‐3‐hydroxyl‐5‐methyl‐4‐isoxazole‐propionate (AMPA) glutamatergic signaling (Ishida et al. 2005). In contrast, tryptamine at 7 mg/L increased brain acetylcholine levels versus control fish here, suggesting that lower brain acetylcholine may correlate with anxiolytic‐ and higher levels—with anxiogenic‐like behaviors (Figures 1 and 2). Notably, higher acetylcholine brain levels are associated with anxiety and depression in both rodent (Mineur et al. 2013) and human studies (Saricicek et al. 2012). Increasing brain acetylcholine levels by another acetylcholinesterase inhibitor, physostigmine, triggers anxiety, and depression in control human subjects and in patients with mood disorders (Janowsky and Overstreet 1990; Risch et al. 1980), whereas blocking acetylcholinesterase activity induces depression/anxiety‐like behavior and social stress in rodents as well, and these effects can be reversed by blocking cholinergic receptors (Mineur et al. 2013). Linking the hyperactive cholinergic system to stress‐related behaviors (Mineur and Picciotto 2010), our present zebrafish findings suggest that trace amines contribute to these CNS effects by modulating central cholinergic neurotransmission.

Finally, we also demonstrated that acute exposure to β‐phenylethylamine (1000 μg/L), tryptamine (7 mg/L), tyramine (15 μg/L) and octopamine (125, 500, and 1500 μg/L) increased whole‐body cortisol levels in zebrafish. In male rats, 10‐day treatment with β‐phenylethylamine elevates plasma corticosterone responses to stress (Kosa et al. 2000), and in Rhesus monkeys, intravenous administration of tryptamine increases plasma cortisol levels (Murphy et al. 1985), supporting the likely evolutionarily conserved link of trace amines to neuroendocrine (glucocorticoid) signaling.

However, the study presents several limitations and potential lines for further research. For example, we did not focus on sex differences in trace amine CNS effects, and this factor merits further scrutiny, as female zebrafish are more anxious (Genario et al. 2020) and may also differ in CNS drug responses from males (dos Santos et al. 2021). Testing a wider range of doses and treatment times (e.g., using both shorter and longer treatments than used here) may also be necessary. We did not explore trace amine effects on the brain monoaminergic (serotonin, dopamine, and noradrenaline) system that plays an important role in CNS regulation and overlaps with the former. Likewise, modulation of other neurotransmitters (including glutamate and gamma aminobutyric acid) may be interesting to assess following trace amine treatment in zebrafish models. Another interesting aspect to consider in future zebrafish studies is to compare several methods of drug administration, including both central and peripheral routes. For example, using more precise and targeted intracerebroventricular (vs. intravenous or intraperitoneal) drug administration may be interesting to perform to complement present studies using water immersion delivery of trace amines. Interestingly, a novel method of intranasal delivery of CNS drugs to adult zebrafish (Galstyan, Kolesnikova, et al. 2025) can be particularly promising to apply, given the well‐reported role of various TAARs as olfactory receptors in the brain. Other limitations of the present study include its main focus on anxiety versus other neurobehavioral domains, such as motor and cognitive aspects, which clearly warrant further scrutiny. Likewise, testing individual differences in fish CNS responses to trace amines, as well as potential strain differences (e.g., especially given mounting evidence of strain behavioral differences in zebrafish assays (van den Bos et al. 2020)), will be relevant.

In addition to acute CNS effects, chronic effects of trace amines also merit further studies. For example, acute treatment with full TAAR1 agonists (e.g., RO5256390) inhibits rat serotonin and dopamine neurons, while chronic treatment increases their excitability in some brain regions (Grinchii et al. 2022). We also did not explore the effect of other trace amines and their metabolites, as well as their action on specific TAARs (e.g., on TAAR1 and other TAARs expressed in zebrafish (Li et al. 2015)), which can help understand which receptors activated by trace amines mediate anxiolytic and anxiogenic‐like CNS effects. Additional putative activity of the four trace amines at other TAARs, non‐TAAR receptors also warrants further studies. Furthermore, as we examined whole‐brain neurochemistry, more nuanced data can be generated from region‐specific analyses of the zebrafish brain.

Taken together, our findings support the importance of trace amines in the regulation of CNS and complex behaviors, and suggest the evolutionary conservation of trace amine neurobehavioral effects across taxa, from zebrafish to mammals (Galstyan, Krotova, et al. 2025). Emphasizing the growing value of zebrafish models for investigating trace amines and TAARs, this highlights the need for further translational cross‐taxon research in this field.

## Author Contributions

**Thalia M. Quintanilha:** conceptualization, investigation, methodology, writing – review and editing, writing – original draft. **Pietra M. Costa:** conceptualization, writing – original draft, investigation, methodology, writing – review and editing. **Ana L. S. Cardoso:** writing – original draft, writing – review and editing, methodology, investigation. **Gabrieli S. Battú:** writing – original draft, writing – review and editing, methodology, investigation. **Leonardo M. Bastos:** investigation, methodology, writing – original draft, writing – review and editing. **Bruno P. dos Santos:** investigation, methodology, writing – original draft, writing – review and editing. **Talise E. Müller:** writing – original draft, writing – review and editing. **Tiago F. de Oliveira:** writing – original draft, writing – review and editing. **Angelo Piato:** writing – original draft, writing – review and editing. **Allan V. Kalueff:** writing – original draft, writing – review and editing, conceptualization. **Murilo S. de Abreu:** conceptualization, investigation, funding acquisition, writing – original draft, writing – review and editing, methodology, supervision, project administration.

## Conflicts of Interest

The authors declare no conflicts of interest.

## Peer Review

The peer review history for this article is available at https://www.webofscience.com/api/gateway/wos/peer‐review/10.1111/jnc.70116.

## Data Availability

The data sets generated during and/or analyzed for the reported study are available from the corresponding authors upon reasonable request.
